# Neutralizing antibodies elicited in rabbits by patient-derived Env trimer immunization

**DOI:** 10.1186/1742-4690-9-S2-P17

**Published:** 2012-09-13

**Authors:** L Heyndrickx, G Stewart-Jones, H Schuitemaker, E Bowles, L Buonaguro, M Jansson, B Grevstad, L Vinner, M Ramaswamy, P Biswas, G Scarlatti, G Vanham, A Fomsgaard

**Affiliations:** 1 Institute of Tropical Medicine, Antwerp, Belgium; 2 Human Immunology Unit, Weatherall Institute of Molecular Medicine, Oxford, UK; 3 Academic Medical Center, University of Amsterdam, Amsterdam, the Netherlands; 4 Istituto Nazionale Tumori "Fond. G. Pascale", Naples, Italy; 5 Department of Laboratory Medicine, University of Lund, Lund, Sweden; 6 Statens Serum Institut, Copenhagen, Denmark; 7 National Institute for Biological Standards and Control, Hertfordshire, UK; 8 DIBIT - San Raffaele Scientific Institute, Milan, Italy

## Background

Eliciting broad cross neutralizing antibodies (bNAb) remains the primary and most challenging goal in HIV-1 vaccine development. So far no vaccine candidate has induced such bNAb. Selecting Env vaccine candidates will require both antigenic and immunogenic optimization and testing in relevant animal models.

## Methods

Based on in-vitro neutralizing activity in serum, patients (n=6, subtype A and B infected) were selected and Env sequences of early HIV-1 variants, still sensitive to autologous neutralization, were used to generate soluble Env as immunogens. Gp140 trimeric proteins were expressed (293T cells) and purified. Rabbits (4/group) were immunized s.c. at weeks 0, 2, 4, 8 with 100µg trimer adjuvanted with cationic CAF01. Control groups received 20µg and 100µg trimer plus/minus CAF01 respectively. Sera collected at weeks 0, 2, 4, 8, 12 and 14 were screened in gp120-IIIB ELISA and IgG was analyzed in the TZMbl neutralization assay.

## Results

All rabbits generated a gp120-IIIB specific IgG response 2 weeks after the first immunization and titers were boosted after each subsequent immunization. IgG titers measured 4 weeks after the last immunization clearly differed between groups (n=5) receiving 100µg/immunization (Geometric mean titer (GMT) : 152.601) and the group receiving 20µg/immunization (GMT : 13.262) or the group omitting CAF01 (GMT : 27.262). Only IgG from rabbits receiving the highest dose and in the presence of CAF01 were able to neutralize Tier 1 pseudoviruses of different subtypes.

Neutralizing activity was detected after the 2nd immunization and was boosted after each immunization. No significant differences were observed between the different trimers.

## Conclusion

Gp140 trimers based on HIV-1 variants of patients with bNAb in serum elicited gp120-IIIB specific IgG and NAb given that enough immunogen was administrated in the presence of CAF01. These results indicate that the development of HIV-1 Env specific NAb is dose dependent and strengthen the rabbit model for HIV vaccine studies.

